# Fe‐Based Nanocrystalline Magnetic Shielding Cylinder with Subfemtotesla‐Level Magnetic Noise

**DOI:** 10.1002/advs.202522435

**Published:** 2026-01-27

**Authors:** Peipei Shen, Danyue Ma, Kun Wang, Shuang Li, Yanan Gao, Pengfei Wang, FuSen Yuan, Hua Chen, Zhuo Wang, Ziling Liu, Meng Xie, Bo Li, Hongbo Zhou, Baoan Sun

**Affiliations:** ^1^ Hangzhou Institute of Extremely‐Weak Magnetic Field Major National Science and Technology Infrastructure Hangzhou China; ^2^ The School of Instrumentation and Optoelectronic Engineering Beihang University Beijing China; ^3^ Hefei National Laboratory Hefei China; ^4^ Institute of Physics Chinese Academy of Sciences Beijing China; ^5^ Institute of Mechanics Chinese Academy of Sciences Beijing China

**Keywords:** Fe‐based nanocrystals, magnetic noise, magnetic shielding, spin‐exchange relaxation‐free

## Abstract

The precision measurement of extremely weak magnetic fields using spin‐exchange relaxation‐free atomic magnetometers demands magnetic shielding system with high shielding efficiency and low magnetic noise. Conventional magnetic shielding system (CMSS) based on *µ*‐metal, however, often exhibits high magnetic noise. Herein, we propose a strategy to overcome this challenge, which is realized by using the Fe‐based nanocrystalline (Fe‐Nano) magnetic shielding cylinder (MSC) as the innermost magnetic shield of CMSS. The Fe‐based nanocrystals (Fe‐Nanos) prepared via the melt‐spinning method followed by a three‐step annealing process exhibit excellent soft magnetic properties with the permeability of 64 000, indicating superior magnetic shielding performance. The average loss factor of the Fe‐Nanos is only 2.4 × 10^−7^, which is significantly lower than that of *µ*‐metal and indicates that Fe‐Nanos has lower intrinsic magnetization noise. The Fe‐Nano MSC with different thicknesses significantly suppresses magnetic noise when used as the innermost magnetic shield of CMSS. In particular, the ten‐layer Fe‐Nano MSC achieves an average magnetic noise of 0.84 fT Hz^−1/2^ in the frequency range of 20–100 Hz. This work provides a lightweight, easily processable, and cost‐effective strategy for the magnetic shielding system with ultra‐low magnetic noise, which will promote its large‐scale application in emerging fields such as biomagnetism and fundamental physics.

## Graphical abstract

Shields with low magnetic noise are achieved by integrating Fe‐based nanocrystalline magnetic shielding cylinders into shielding systems based on *μ*‐metal. Benefiting from high permeability and low loss factor, Fe‐based nanocrystalline magnetic shielding cylinders effectively suppress magnetic noise. Notably, the magnetic shielding cylinder with the ten‐layer reaches 0.84 fT Hz^−1/2^, supporting ultra‐sensitive magnetic field detection.

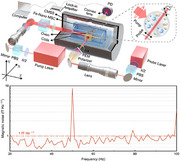

## 1 Introduction

Advanced techniques for measuring extremely weak magnetic fields, including spin‐exchange relaxation‐free (SERF) atomic magnetometer (AM), superconducting quantum interference device (SQUID), and nitrogen‐vacancy (NV) centers in diamond, have attracted considerable interest owing to their ability to detect magnetic fields with high sensitivity [1, 2, 3]. In particular, the developments of AM with ultrahigh‐sensitivity have made significant progress in fundamental physics research (FPR), serving as powerful tools for probing exotic interactions and dark matter [4, 5]. AM is also employed in nondestructive material characterization [6]. What's more, high‐resolution imaging of the extremely weak biomagnetism generated by the human body can provide important information for health monitoring and medical treatment. Although SQUID has made some achievements in biomagnetism measurements, their widespread application is hindered [7]. The primary drawbacks of SQUID arise from their high cost and demanding maintenance requirements. In the past few decades, significant progress has been made in the study of adult magnetocardiography (aMCG) and magnetoencephalography (MEG) with the development of the multichannel AM and wearable AM systems [8, 9]. Compared with the SQUID employed as sensors for biomagnetism, the SERF AM operates without reliance on cryogenic conditions and detects vector magnetic fields, which is beneficial for saving precious liquid helium resources and obtaining an overall higher signal strength [10].

The precision measurements of extremely weak magnetic fields based on SERF AM require low magnetic noise and near zero magnetic field environment [11, 12, 13], which is typically achieved through the magnetic shielding system with high shielding efficiency. Currently, the conventional magnetic shielding system (CMSS), which has a nested cylindrical structure, consists of a single‐layer aluminum alloy and multilayer *µ*‐metal [14]. While CMSS can effectively shield against external magnetic fields, the innermost magnetic shield made of *µ*‐metal introduces inherently high magnetic noise due to its low resistivity. The high magnetic noise is a critical factor that seriously affects the precision measurement of biomagnetism in the brain, muscles, and nerve fibers [15, 16]. Therefore, researchers have explored ferrite magnetic shielding cylinder (MSC), Fe‐based and Co‐based amorphous/nanocrystalline MSC as the innermost magnetic shield of CMSS to suppress magnetic noise generated by *µ*‐metal. Ferrite MSC, when employed as the innermost magnetic shield of the CMSS, can significantly suppress magnetic noise [17]. However, the issues of limitations in volume and lengthy manufacturing cycles remain unsolved at present.

Fe‐based and Co‐based amorphous/nanocrystalline alloys combine the characteristics of high permeability, high resistivity, and flexibility, making them suitable as magnetic shields with low magnetic noise [18]. Compared with the Co‐based amorphous alloy (Co‐based AA), Fe‐based nanocrystals (Fe‐Nanos) have excellent soft magnetic properties and the advantage of low cost. In the past few years, there have been some reports about Fe‐based nanocrystalline (Fe‐Nano) MSC as the innermost magnetic shield of CMSS, but their magnetic noise remains in the range of tens of fT Hz^−1/2^ (1 fT = 10^−15^ T) [19, 20], which is still difficult to meet the requirements for precision measurement of extremely weak magnetic fields. Therefore, there is an urgent need to manufacture MSC with ultralow magnetic noise as the innermost magnetic shield of CMSS through a short‐time, low‐cost, and high‐efficiency process to meet the demands of high‐precision weak magnetic field measurement.

This research aims to significantly suppress the magnetic noise of CMSS by using the Fe‐Nano MSC as the innermost magnetic shield. In this work, the Finemet‐type Fe‐Nano ribbon, which has soft magnetic properties and high permeability, was prepared through a three‐step annealing method without applying magnetic field. Additionally, the Fe‐Nano ribbon with 18–22 µm thickness and 50 mm width exhibits ultralow magnetic noise characteristics at low frequencies in weak magnetic field environments, according to the power dissipation theory. Subsequently, the multilayer Fe‐Nano magnetic shielding cylinders (MSCs) with different thicknesses were fabricated using the Fe‐Nano ribbons, and their magnetic noise as the innermost magnetic shield of CMSS was characterized using a SERF AM with a sensitivity of 1.0 fT Hz^−1/2^. It was found that the magnetic noise suppression effect significantly improves with the increase in thickness. Finally, the average magnetic noise of the ten‐layer Fe‐Nano MSC was further measured to be 0.84 fT Hz^−1/2^ using a SERF magnetic field measurement setup (MFMS) with a sensitivity of 0.4 fT Hz^−1/2^ [21], which is about 96% lower than the results reported in previous literature [19, 20]. This work thus ensures the compatibility of soft magnetic properties and low magnetic noise of the Fe‐Nano ribbon, and the advantages of economy and high‐efficiency process will facilitate their large‐scale application in emerging fields such as biomagnetism measurements, nuclear spin gyroscope, electric dipole moment, and dark matter detection [4, 22, 23, 24, 25].

## 2 Results

### 2.1 Structure Analysis

**FIGURE 1:**
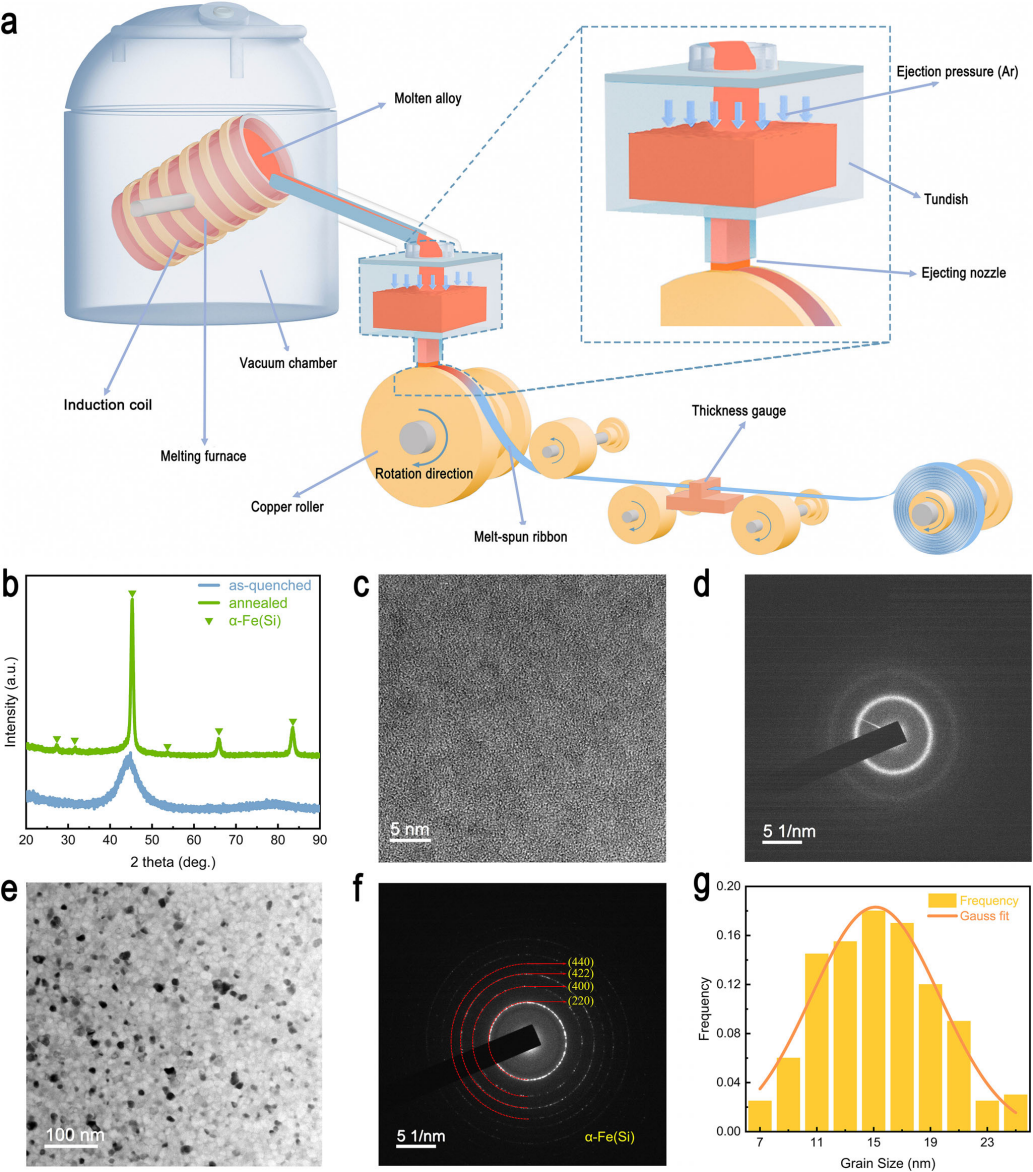
Manufacturing and structure characterization of the Fe‐based amorphous and nanocrystalline alloy ribbon. a) Schematic diagram of the single‐roller melt‐spinning method. b) X‐ray diffraction (XRD) patterns of the as‐quenched and annealed Fe‐based ribbons at room temperature. c) The high‐resolution transmission electron microscope (HRTEM) image of the as‐quenched Fe‐based ribbon. d) The corresponding selected area electron diffraction (SEAD) pattern for the as‐quenched Fe‐based ribbon. e) TEM image of the annealed Fe‐based ribbon. f) Selected area electron diffraction patterns of the annealed Fe‐based ribbon. g) The statistical distribution of grain size in Figure 1e.

The as‐quenched Fe‐based amorphous ribbon with a width of 50 mm was manufactured by the single‐roller melt‐spinning method, as shown in Figure 1a. The cooling rate of this method reaches ∼10^6^ K/s, which is sufficient to suppress the nucleation and prevent crystallization. The as‐quenched ribbons manufactured by this method could reach lengths of tens of thousands of meters, with a thickness of 18–22 µm. For subsequent annealing and magnetic properties tests, the Fe‐based amorphous ribbons were rewound into cores with an outer diameter of 100 mm and an inner diameter of 80 mm (Figure S1) using a core‐winding apparatus. The Fe‐Nano ribbons with a single α‐Fe(Si) phase were obtained from Fe‐based amorphous cores via a three‐step annealing process. The detailed manufacturing process is summarized in the Experimental Section.

Figure 1b presents the X‐ray diffraction (XRD) patterns of the as‐quenched and annealed Fe‐based ribbons in the 2 theta angular range of 20–90^○^. The as‐quenched Fe‐based ribbon only exhibits a smooth diffraction hump in the 2 theta angular range of 34–53° without distinct crystalline peaks observed, demonstrating that the structure is fully glassy. Besides, the surface morphology of the as‐quenched Fe‐based ribbon was observed with a scanning electron microscope (SEM) in the secondary electron (SE) imaging modes. The working distance was set to approximately 8–10 mm, and images were captured at different magnifications to assess surface roughness between the bottom surface (copper‐roller‐contacted side) and top surface (air‐contacted side). The SEM images with different scales for the bottom surface and the top surface of as‐quenched Fe‐based ribbons are presented in Figures S2 and S3. Based on these results, the bottom surface is noticeably rougher than the top surface, which is attributed to the direct contact between the ribbon's bottom surface and the copper roller during the melt‐spinning process. Compared with the XRD result of the as‐quenched ribbon, a series of sharp peaks only corresponding to α‐Fe(Si) are detected for the annealed ribbon, suggesting that it possesses a single‐phase nanocrystalline structure.

The transmission electron microscopy (TEM) characterizations were also performed to study the microstructure of the as‐quenched and annealed Fe‐based ribbons. As shown in Figure 1c, the high‐resolution TEM (HRTEM) image of the as‐quenched Fe‐based ribbon confirms the lack of periodic lattice fringes, indicating the structural homogeneity of the amorphous phase. The amorphous nature was also confirmed by the selected area electron diffraction (SAED) pattern (displayed in Figure 1d), which exhibits diffuse diffraction halos. In contrast, the bright‐field TEM (BF‐TEM) image in Figure 1e displays finely dispersed nanocrystalline regions distributed randomly with almost no amorphous regions for the annealed Fe‐based ribbon. The SAED pattern acquired from the nanocrystalline regions exhibits diffraction rings of the (220), (400), (422), and (440) of the α‐Fe(Si) phase (as proved in Figure 1f), which is in agreement with the XRD result. To evaluate the crystallization behavior of the annealed Fe‐based ribbons, the statistical analysis of the grain size distribution was conducted based on the BF‐TEM images. Approximately 200 individual nanocrystals were measured, and their grain size distribution was analyzed using *Nanomeasure* software. Figure 1g presents the grain size distribution histogram of approximately 200 individual nanocrystals. The results show a relatively narrow grain size distribution, with grain diameters predominantly ranging between 11 and 19 nm. The average grain size is determined to be only 15.4 nm, indicating a well‐controlled nucleation and growth process. Such an ultrafine microstructure is advantageous for achieving soft magnetic properties in Fe‐Nanos.

### 2.2 Magnetic Performance and Magnetic Noise Mechanism

**FIGURE 2:**
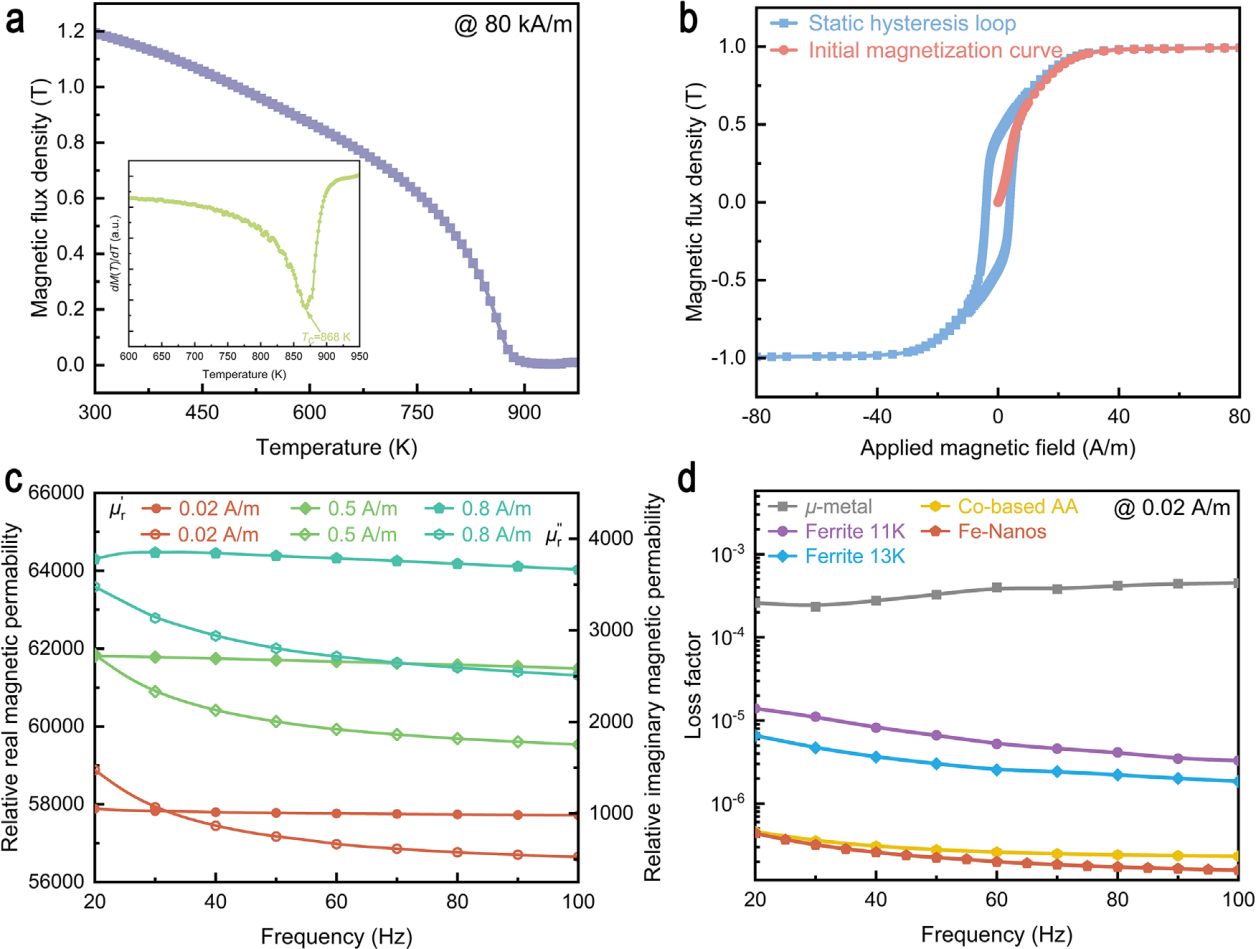
Magnetic properties and loss factor of the Fe‐based nanocrystalline ribbon. a) The saturation magnetization as a function of temperature (*M*–*T* curves) of the Fe‐based nanocrystalline alloy ribbon under a magnetic field of 80 kA/m. The inset presents the (*dM*(*T*)/*dT*)–*T* curve of Fe‐based nanocrystalline alloy ribbon. b) Initial magnetization curve and hysteresis loop of Fe‐based nanocrystalline core. c) Real part (µ′_*r*_, Left Axis) and imaginary part (µ″_*r*_, Right Axis) of the relative complex permeability vs frequency (from 20 to 100 Hz) for Fe‐based nanocrystalline core under different magnetic fields. d) Frequency dependence of the loss factor for different soft magnetic materials under a 0.02 A/m magnetic field evaluated by µ″_*r*_/µ′_r_^2^.

To determine the Curie temperature (*T*_c_) of the Fe‐Nano ribbon, the saturation magnetization (*M*_s_) was measured over a temperature range from 300 to 975 K. As shown in Figure 2a, the *M*_s_ is 1.2 T at 300 K and decreases monotonically with increasing temperature, reflecting the gradual weakening of long‐range ferromagnetic order. Notably, the *T*_c_ of the Fe‐Nano ribbons is determined as 868 K (as demonstrated in the inset of Figure 2a), based on the inflection point of the first derivative of the magnetization with temperature. Such a high *T*_c_ indicates that the Fe‐Nanos in this work remain operational in high temperatures, without being adversely affected by the thermal radiation from the SERF AM. Figure 2b displays the magnetization curve of the Fe‐Nano core. Based on the initial magnetization curve, the permeability *µ*_r_ is 64 000, which is significantly better than that of the ferrites [26]. Such a high permeability indicates excellent potential for effective magnetic shielding applications for the SERF AM. The corresponding hysteresis loop exhibits a narrow profile with a low coercivity of 3.8 A/m, which is a typical characteristic of soft magnetic property. The soft magnetic performance of Fe‐Nanos is closely related to ultrafine grain structure, as described by the random anisotropy model [27].

In the application of extremely weak magnetic field measurement performed with SERF AM, CMSS must not only suppress external magnetic interference but also exhibit intrinsically low magnetic noise, which would otherwise limit the achievable measurement precision of the extremely weak magnetic fields. Therefore, ideal magnetic shielding materials should combine high magnetic permeability with an intrinsically low magnetic noise. Based on the fluctuation‐dissipation theorem, the intrinsic magnetic noise of magnetic shielding materials can be separated into two primary contributions: magnetization noise (*δB*_magn_) and Johnson noise (*δB*_eddy_), described by Equations (1) and (2) [17]: 
(1)

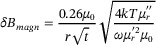

(2) $$\delta B_{eddy}=\frac{\mu_{0}\sqrt{t}}{4r}\sqrt{3kT\sigma }C\left(\mu \right)$$

here μ_0_ denotes vacuum permeability, *k* is the Boltzmann constant, and *T* is the temperature. The angular frequency is *ω* = 2π*f*. The $\mu_{r}^{\prime }$ and 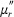 represent the real part and imaginary part of the relative complex permeability, *σ* is the electrical conductivity. The *r* and *t* are the radius and the thickness of the magnetic shield. For *µ*′*/µ*0 ≫ 1, the factor *C(µ)* approaches a constant value of approximately 0.7. Due to the close correlation between *δB*_magn_ and the complex permeability expressed by Equation (1), we performed systematic measurements of the inductance (*L*_s_) and resistance (*R*_s_) of the Fe‐Nano core over the frequency range of 20 to 100 Hz under different magnetic fields. These electrical parameters were then used to determine the $\mu_{r}^{\prime }$ and 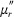 using Equations (3) and (4) [28]: 

(3)

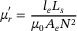



(4)

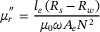

where *l*_e_ and *A*_e_ describe the effective magnetic path length and cross‐sectional area of the toroid specimen, respectively. While *R*_w_ and 𝑁 represent the DC resistance and the number of turns of the measuring coil.

Figure 2c illustrates the frequency dependence of the $\mu_{r}^{\prime }$ and 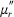 for the Fe‐Nano core measured under different magnetic fields. The results indicate that there is almost no change in $\mu_{r}^{\prime }$ within the frequency range of 20–100 Hz, indicating an essentially frequency‐independent magnetic behavior within this low‐frequency regime. Notably, slight increase in $\mu_{r}^{\prime }$ is observed as the applied magnetic field increases from 0.02 A/m to 0.8 A/m, which is attributable to enhanced domain wall mobility. In contrast, the 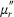 of the core exhibits a significant decrease with increasing frequency and decreasing magnetic field over the same frequency range. The combination of stable $\mu_{r}^{\prime }$ and suppressed 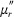 implies that the intrinsic noise, governed by magnetization noise mechanisms, is reduced as the magnetic field decreases.

With the development of SERF AM, the choice of magnetic shielding materials is a critical factor in determining device performance. Ferrites and *µ*‐metal (*e.g*., Mn–Zn ferrite, 1J85) have become the most commonly used materials for the innermost magnetic shield due to their ability to suppress external magnetic disturbances. As described by Equation (1), the intrinsic *δB*_magn_ of the soft magnetic materials is strongly related to their loss factor (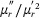). To assess the potential of Fe‐Nanos as low magnetic noise shielding materials, we analyzed their frequency dependence of loss factor at 0.02 A/m and compared with other magnetic shielding materials, which is presented in Figure 2d. The frequency dependence of the $\mu_{r}^{\prime }$ and 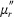 for *µ*‐metal, Ferrite 11 K, Ferrite 13 K, and Co‐based AA are presented in Figures S4–S7.

As shown in Figure 2d, the loss factor of *µ*‐metal slightly increases with rising frequency under a magnetic field of 0.02 A/m, while the loss factors of Ferrite 11 K, Ferrite 13 K, Co‐based AA, and Fe‐Nanos all decrease with increasing frequency in the 20–100 Hz range. Specifically, the loss factor of *µ*‐metal is approximately 10^−4^, while the loss factor of ferrite 11 K is approximately 10^−5^; Ferrite 13 K exhibits a lower loss factor of approximately 3.2  × 10^−6^, and both Co‐based AA and Fe‐Nanos display loss factors within the order of 10^−7^. Furthermore, the Fe‐Nano show an average loss factor of only 2.4  ×  10^−7^, which is lower than that of Co‐based AA and indicates that the Fe‐Nanos possess a substantially lower intrinsic magnetization noise. The notable reduction in the loss factor demonstrates the considerable potential of Fe‐Nanos for ultra‐low magnetic noise magnetic shielding applications, especially in extremely weak magnetic field detection scenarios.

### 2.3 Demagnetization Analysis

Remanence arising from the hysteresis of soft magnetic materials contributes to the residual magnetic field within the magnetic shielding space and degrades the precision of SERF AM. Therefore, it is essential to understand the demagnetization characteristics of Fe‐Nanos. The Jiles‐Atherton (JA) model, based on the theory of domain wall motion, offers a physical description of ferromagnetic hysteresis. The differential equations of the JA model are expressed as follows:

(5) $$M=M_{irr}+M_{rev}$$

(6) $$H_{e}=H+\alpha M$$

(7) $$M_{an}=M_{s}\;\left[\coth\left(\frac{H_{e}}{a}\right)-\left(\frac{a}{H_{e}}\right)\right]$$

(8) $$\frac{dM_{irr}}{dH}=\frac{M_{an}-M_{irr}}{\delta k_{loss}/\mu_{0}-\alpha \left(M_{an}+M_{rev}\right)}$$

(9) $$M_{rev}=c\left(M_{an}-M_{irr}\right)$$

where the total magnetization *M* is the sum of the irreversible component *M*_irr_ and the reversible component *M*_rev_, which is associated with the anhysteretic magnetization *M*_an_. The effective magnetic field *H*_e_ represents the local magnetic field acting on a single magnetic moment. *M*_s_ represents the saturation magnetization of the magnetic material. The parameters *α* and *a* reflect the mean‐field interaction between neighboring domains and the density of domain walls. In addition, *k_loss_* and *c* describe the loss coefficient and the magnetization reversibility. When the magnetic field *H* increases in the positive direction, the parameter *δ* is taken as+1, otherwise it takes −1.

**FIGURE 3:**
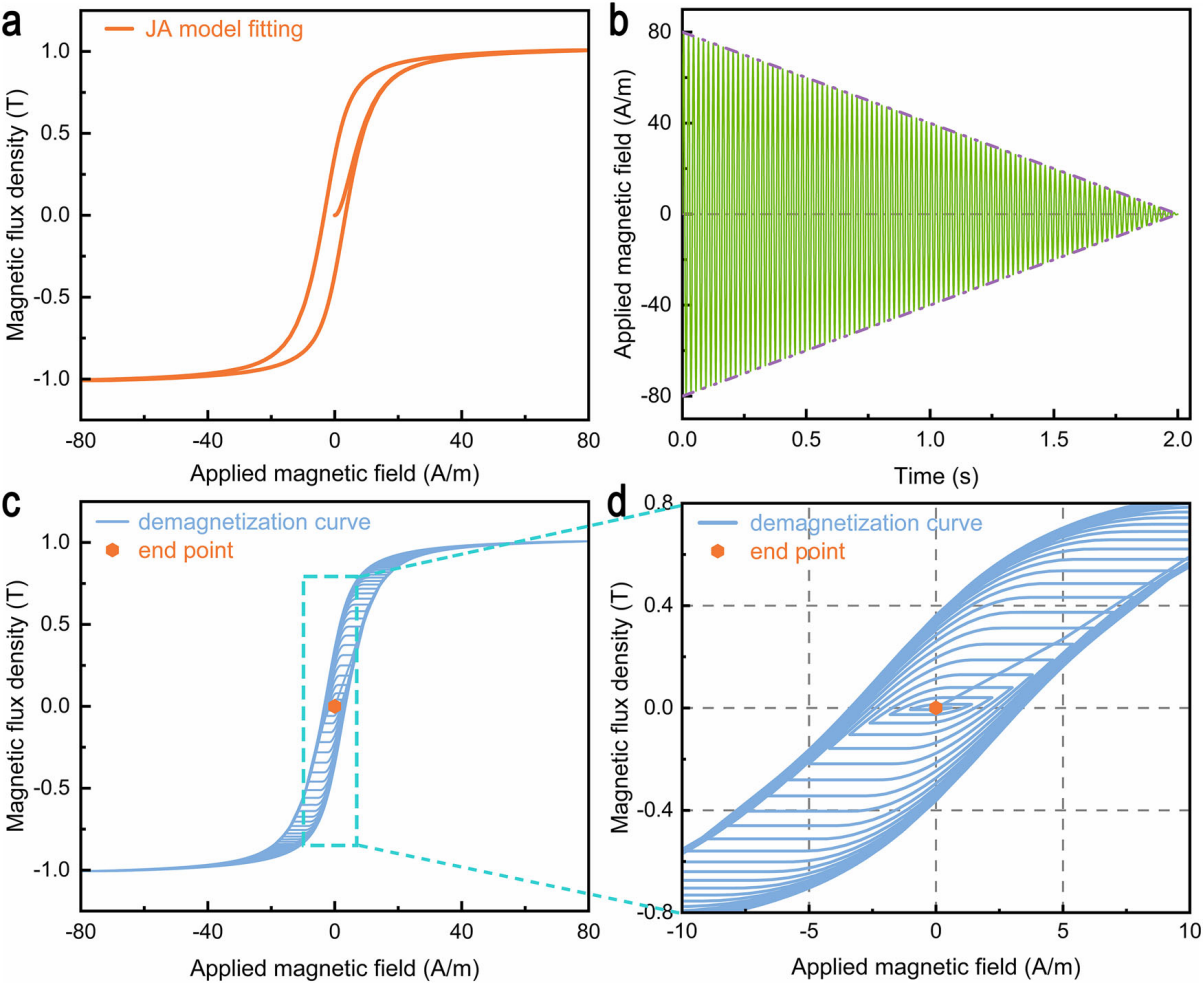
The simulation of the demagnetization behavior of Fe‐based nanocrystalline ribbon under a first‐order envelope field. a) Calculated static magnetization curve under the applied field of 80 A/m for Fe‐based nanocrystalline ribbon, obtained using optimized Jiles‐Atherton model parameters. b) Time‐dependent demagnetization field based on the first‐order envelope function with the initial field amplitude *H*_0_ = 80 A/m. c) Demagnetization curve of Fe‐based nanocrystalline ribbon with its endpoint under a 50 Hz sine wave. d) The demagnetization curve was enlarged within the box area in Figure 3c.

To accurately predict the remanence of Fe‐Nanos after degaussing, the JA model was employed to fit the hysteresis loop displayed in Figure 2b, and to analyze the associated demagnetization process. Due to the JA model's intrinsic nonlinearity, the particle swarm optimization algorithm was utilized to iteratively extract the optimal parameters, which were listed in Table 1. Figure 3a presents the hysteresis loop fitted by using the optimized JA model's parameters. The close agreement between the fitted and experimental results suggests that the parameter set in Table 1 reliably reflects the static magnetic behavior of the Fe‐Nanos.

**TABLE 1:** Parameters of the JA model for Fe‐Nanos used in this work.

Parameter	Value	Unit
*M*_s_	8.29 × 10^5^	A/m
*α*	1.03 × 10^−8^	1
*a*	2.57	A/m
*k*_loss_	3.71	A/m
*c*	1 × 10^−7^	1

The demagnetization field, which is a time‐dependent magnetic field, was modulated by a first‐order envelope function (see the “Demagnetization model” section in Methods). Here, the initial field amplitude *H*_0_ and total demagnetization duration *T*_degauss_ are set to 80 A/m and 2 s, respectively. As shown in Figure 3b, the demagnetization field exhibits a linearly decaying sinusoidal profile, characterized by a linear reduction in amplitude over time while maintaining constant oscillation frequency, which promotes the progressive randomization of magnetic domain orientations.

The magnetization evolution of Fe‐Nanos during the demagnetization process is simulated using the demagnetization field shown in Figure 3b and the optimized JA model's parameters. The demagnetization loops with the endpoint, shown in Figure 3c, demonstrate a clear convergence of the magnetization toward the anhysteretic curve, indicating that the domain wall orientation gradually becomes random throughout the demagnetization process. It is worth noting that, as shown in Figure 3d, the remanence at the end of the demagnetization is effectively suppressed to near zero level, which confirms that the demagnetization field employed in this work is capable of achieving deep demagnetization in Fe‐Nano materials. The identified degaussing parameters will be used to guide the demagnetization experiment to realize optimal demagnetization.

### 2.4 Fe‐Based Nanocrystalline Magnetic Shielding Cylinder

**FIGURE 4:**
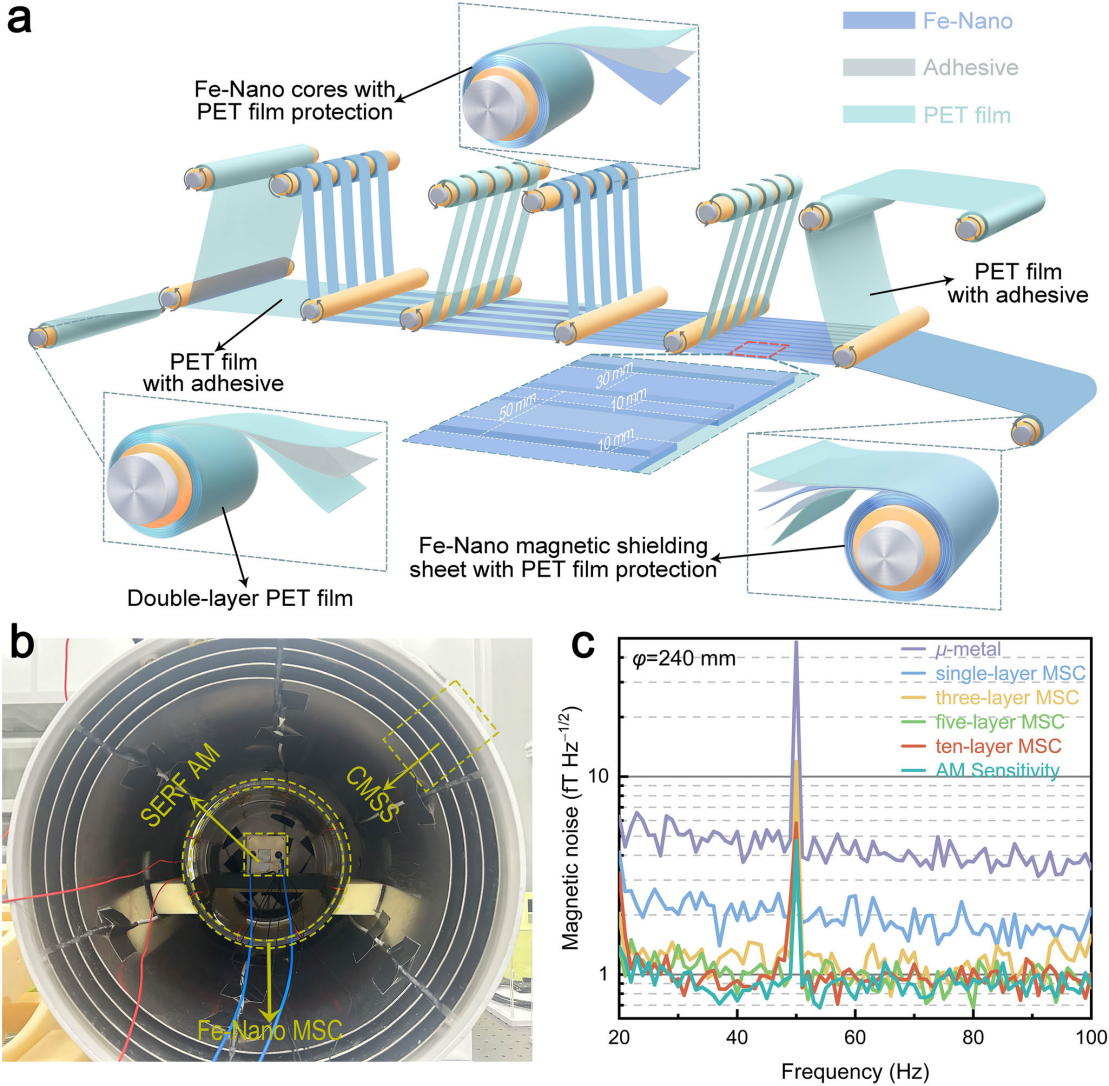
Fabrication process of Fe‐based nanocrystalline magnetic shielding sheets and noise characterization of Fe‐based nanocrystalline magnetic shielding cylinders. a) Schematic illustration of the fabrication process of magnetic shielding sheets using Fe‐based nanocrystalline ribbons. The top inset shows the structure of Fe‐based nanocrystalline ribbon with the PET film protection. The bottom‐left inset presents the double‐layer PET film structure with a width of 410 mm. The bottom‐middle and bottom‐right inset illustrates the 10 mm overlap region during the splicing process and the layered structure of the magnetic shielding sheet. For clarity, the dimensions of each part in the figure were not strictly drawn according to the real scale. b) Photograph of the experimental setup for noise measurement with SERF AM. c) Magnetic noise spectrum of the Fe‐based nanocrystalline magnetic shielding cylinder and *µ*‐metal magnetic shield measured by the SERF AM with a sensitivity of 1.0 fT Hz^−1/2^.

Fe‐Nano ribbons typically exhibit brittleness and fracture, which severely limit their structural reliability in practical applications. To overcome this limitation, a mechanical reinforcement strategy was implemented by laminating polyethylene terephthalate (PET) film onto the top surface of the Fe‐Nano ribbons using an adhesive with a thickness of approximately 3 µm, which enhances their overall mechanical strength and surface flatness during forming processes (top inset in Figure 4a). Multiple PET‐laminated Fe‐Nano ribbons were carefully spliced together using an insulating adhesive and sheared to produce magnetic shielding sheet with a uniform width of 390 mm. In addition, an overlapping area of 10 mm wide was introduced between adjacent ribbons, which effectively suppressed magnetic flux leakage at the seams and thereby preserved the continuity and overall performance of the magnetic shielding sheet (for details see Figure 4a).

The Fe‐Nano MSC is composed of a cylindrical main body and two endcaps. The main body was fabricated by tightly winding shielding sheet onto a non‐magnetic acrylic cylindrical frame with a diameter of 240 mm, whose design follows a structure similar to that reported in previous literature [29]. The thickness of the main body could be precisely tailored by adjusting the number of winding layers of the shielding sheet. Previous studies have demonstrated that the effectiveness of magnetic noise suppression is strongly dependent on the thickness of the MSC [29]. In addition, each of the Fe‐Nano magnetic shielding sheets in the Fe‐Nano MSC is separated by PET films and insulating adhesives. In this case, the intrinsic magnetic noise generated in the outer Fe‐Nano magnetic shielding sheet of MSC can be effectively attenuated by the inner Fe‐Nano magnetic shielding sheet. As a result, the magnetic noise inside the shields is primarily determined by the intrinsic noise of the innermost Fe‐Nano magnetic shielding sheet when the Fe‐Nano MSC completely suppresses the magnetic noise generated by the *µ*‐metal. Therefore, four types of Fe‐Nnano MSC with different thicknesses—namely, single‐layer, three‐layer, five‐layer, and ten‐layer—were designed for comparative investigation. Meanwhile, the endcaps were fabricated from circular magnetic shielding sheets with the same number of layers as the cylindrical body. In addition, each endcap contained a central hole with a diameter of 50 mm, while the body was equipped with four uniformly distributed holes with a diameter of 40 mm to allow the pump and probe laser beams to pass through during subsequent experiments. Furthermore, demagnetization coils were uniformly wound on both surfaces of the MSC to avoid interference of remanence (Figure S8). Driven by an alternating current of gradually decreasing amplitude, these coils generated a decaying time‐varying magnetic field, thereby effectively randomizing the orientation of magnetic domains and eliminating residual magnetic interference. Since the resistivity of Fe‐Nanos is approximately 138 µΩ cm at room temperature (Figure S9), the *δB*_eddy_ can be estimated to be around 0.78 fT Hz^−1/2^, according to Equation (2).

The suppression effect of magnetic noise of the Fe‐Nano MSCs with different thicknesses was evaluated using a SERF AM with a sensitivity of 1.0 fT Hz^−1/2^ (shown in Figure 4b). The basic principle of the SERF AM is as follows: A vapor cell containing a droplet of Rubidium metal and injected with N_2_ as both quenching and buffer gas is heated to approximately 433 K through electrical heating. The high rubidium vapor density, increased by the heating process, and the near‐zero magnetic field environment enable Rubidium atoms to operate in the SERF regime. A circular‐polarized pump laser passes through the cell and optically polarizes the Rubidium atoms. Meanwhile, a linear‐polarized probe beam is directed orthogonally to the pump beam to monitor the Rubidium atomic polarization. When an external field is applied, the polarization of the Rubidium atoms changes, causing a rotation in the polarization of the probe laser. Finally, the polarization of the probe laser is detected by the photoelectric detector [30, 31]. Figure 4c presents the suppression effect of magnetic noise achieved by Fe‐Nano MSCs when employed as the innermost shields within a CMSS. Notably, abnormal noise occurs near 50 Hz due to power frequency interference. Without the Fe‐Nano MSC as the innermost magnetic shield, the average magnetic noise of the *µ*‐metal magnetic shield is approximately 4.3 fT Hz^−1/2^. In addition, it is evident that when the single‐layer Fe‐Nano MSC is employed as the innermost magnetic shield, the average magnetic noise in the range of 20–100 Hz decreases to approximately 2.0 fT Hz^−1/2^, which confirms that even the single‐layer Fe‐Nano MSC provides effective attenuation of the magnetic noise originating from the *µ*‐metal. Moreover, the noise suppression effect is further enhanced with increasing MSC's thickness. In particular, the five‐layer and ten‐layer Fe‐based MSCs achieve an average noise level of less than 1.1 fT Hz^−1/2^ within the same frequency range, which demonstrates that the multi‐layer Fe‐based MSC establishes an ultralow magnetic noise environment. It should be noted that the magnetic noise of the ten‐layer Fe‐based MSC approaches the inherent sensitivity limit of the SERF AM with a sensitivity of 1.0 fT Hz^−1/2^. In order to further accurately measure the inherent magnetic noise of the ten‐layer Fe‐Nano MSC beyond the detection limit of SERF AM, the MFMS with a sensitivity of 0.4 fT Hz^−1/2^ was subsequently employed to characterize the magnetic noise of the ten‐layer Fe‐Nano MSC [21].

**FIGURE 5:**
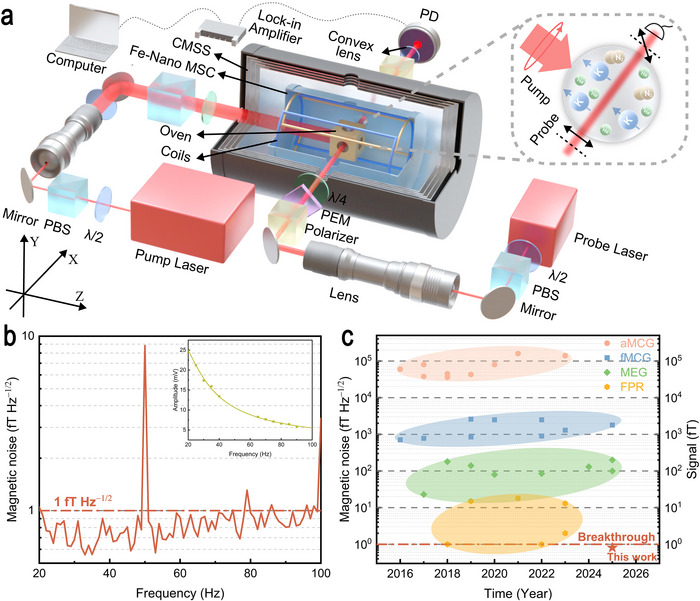
Characterization of magnetic noise in Fe‐based nanocrystalline magnetic shielding cylinder using SERF magnetic field measurement setup and its potential in biomagnetism and fundamental physics research. a) The schematic diagram of the magnetic field measurement setup based on the SERF effect of K atoms. *λ*/2: half‐wave plate; *λ*/4: quarter‐wave plate; PBS: polarization beam splitter; PEM: photoelastic modulator; PD: photodiode; b) Magnetic noise spectrum of the ten‐layer Fe‐based nanocrystalline magnetic shielding cylinder measured by the magnetic field measurement setup. The inset shows the frequency response. c) Comparison of biomagnetism signal in adult and fetal magnetocardiography (aMCG, fMCG) [34, 35, 36, 37, 38, 39, 40, 41, 42, 43, 44, 45, 46, 47, 48, 49, 50, 51], magnetoencephalography (MEG) [52, 53, 54, 55, 56, 57, 58, 59, 60], and the sensitivity of SERF atomic magnetometers in fundamental physics research (FPR) [4, 5, 61, 62, 63, 64], highlighting the broad application potential of Fe‐based nanocrystalline magnetic shielding cylinder.

The schematic diagram of MFMS based on the SERF effect of potassium (K) atoms is shown in Figure 5a. To suppress the interference of mechanical vibrations, the MFMS was placed on a vibration‐isolated platform. The sensitive element is a spherical vapor cell with a diameter of 30 mm, containing a droplet of potassium metal, 30 Torr N_2_ as quenching gas, and 1300 Torr ^4^He as buffer gas. During the experiment, the vapor cell was positioned at the center of the CMSS and maintained at 473 K using a temperature‐controlled oven equipped with non‐magnetic electrical heating film [32, 33]. The CMSS, which consists of an aluminum and a four‐layer *µ*‐metal shield, was used to attenuate external magnetic fields to below 1 nT (1 nT = 10^−9^ T). Three‐axis compensation coils were employed to further null residual fields and generate controlled magnetic fields for calibration and measurement. The ten‐layer Fe‐Nano MSC was positioned between the compensation coils and the CMSS to further suppress magnetic noise. After demagnetization, the residual magnetic fields of the ten‐layer Fe‐based nanocrystalline shielding cylinder were below 0.8, 0.5, and 0.7 nT along the X, Y, and Z directions, respectively. The pump laser beam was generated by a distributed Bragg reflector (DBR) laser with a tapered amplifier tuned to the center of the K D1 line. It was expanded to a 30 mm diameter and circularly polarized. When directed along the *z*‐axis through the vapor cell, the pump beam polarized the K atoms and enabling the vapor to respond to external magnetic fields. Meanwhile, the probe laser beam, emitted from a separate DBR laser, was tuned to approximately 100 GHz off the D1 line of K and expanded to 3 mm. The probe beam, linearly polarized after a polarizer and modulated at 50 kHz by a photoelastic modulator (PEM), was transmitted along the *x*‐axis through the vapor cell and an analyzer before being detected by a photodetector (PD). The resulting signal is demodulated using a lock‐in amplifier and subsequently digitized for data acquisition and analysis with a sampling rate of 899.5 Hz.

Figure 5b presents the magnetic noise spectrum of the ten‐layer Fe‐Nano MSC obtained using the MFMS. The frequency response recorded for 30 s under the reference signal of 10 pTrms is illustrated in the inset of Figure 5b. The fast Fourier transform was performed on the collected signal, and the averaged power spectral density value at the 1‐Hz bin (the average power spectral density within the 0.5–1.5 Hz band) was calculated. Magnetic noise of the Fe‐Nano MSC was then calculated by dividing the power spectral density by the frequency response. As can be observed from Figure 5b, the average magnetic noise of the ten‐layer Fe‐Nano MSC is only 0.84 fT Hz^−1/2^, indicating a reduction of roughly 96% compared to the results reported in previous literature [19, 20]. In order to highlight the broad application potential of the Fe‐Nano MSC developed in this work, we have summarized recent some studies on biomagnetism signals, including adult magnetocardiography (aMCG) [34, 35, 36, 37, 38, 39, 40, 41, 42, 43], fetal magnetocardiography (fMCG) [44, 45, 46, 47, 48, 49, 50, 51], and magnetoencephalography (MEG) [52, 53, 54, 55, 56, 57, 58, 59, 60], as well as the sensitivity of SERF AM required in fundamental physics research (FPR) [4, 5, 61, 62, 63, 64], as illustrated in Figure 5c. Previous studies have reported that aMCG signals typically reach a magnitude of several tens of pT (1 pT = 1000 fT), whereas fMCG signals are only a few pT. By contrast, MEG signals are much weaker, usually only tens to hundreds of fT, while atomic magnetometers employed in FPR generally exhibit sensitivity better than 20 fT Hz^−1/2^. With the ultra‐low magnetic noise of 0.84 fT Hz^−1/2^, the Fe‐Nano MSC provides an ideal low‐noise environment for research in these fields. According to the fluctuation‐dissipation theorem, the intrinsic magnetic noise is inversely proportional to the diameter of the MSC, which suggests that the Fe‐Nano MSC with a diameter of 1000 mm could further reduce the noise to approximately 0.2 fT Hz^−1/2^. Beyond its ultra‐low magnetic noise performance, the Fe‐Nano MSC also possesses additional advantages such as low cost, lightweight, and flexibility, which further enhance its applicability in specialized scenarios.

## 3 Conclusion

In summary, the Fe‐Nano ribbons developed in this work exhibit excellent soft magnetic properties and low loss factor, which ensures high shielding performance while maintaining intrinsically ultra‐low magnetic noise. When employed as the innermost magnetic shield of conventional magnetic shielding system, the Fe‐based nanocrystalline magnetic shielding cylinder exhibits a significant enhancement in magnetic noise suppression with increasing shield thickness. In particular, the ten‐layer Fe‐based nanocrystalline magnetic shielding cylinder achieves an average magnetic noise as low as 0.84 fT Hz^−1/2^, which is approaching the noise level of ferrite magnetic shielding cylinder. This work provides a low‐cost and high‐efficiency route for designing the magnetic shielding system with ultralow magnetic noise, which is expected to support research in biomagnetism, fundamental physics, and other frontier fields requiring the detection of extremely weak magnetic fields.

## 4 Experimental Section

### 4.1 Materials Fabrication

The as‐quenched Fe‐based amorphous ribbon was prepared via a two‐step procedure. First, the chemically homogeneous molten alloy was obtained by induction melting a mixture of industrial‐grade raw metals and metalloids (purity ≥ 99.5 wt.%) for at least 30 min in vacuum (<50 Pa) with a melting furnace. Subsequently, the molten alloy was poured into a tundish with excellent thermal insulation properties to ensure a stable melt temperature and spun into Fe‐based amorphous ribbon with a width of 50 mm using the single‐roller melt‐spinning method in a controlled Ar atmosphere. During the process, the linear speed of the copper roller with a diameter of 980 mm was set to 30 m/s. A jet of the molten alloy was ejected through the nozzle of the tundish onto the surface of the rotating copper roller, enabling rapid cooling of the molten alloy to form the as‐quenched ribbon. The thickness of the Fe‐based amorphous ribbon was controlled to be 20±2 µm by adjusting the Ar pressure and the distance between the ejecting nozzle and the copper roller. The as‐quenched ribbon was then continuously wound onto a take‐up roller with a diameter of 350 mm for collection (for details see Figure 1a).

### 4.2 Annealing Process

As demonstrated in the previous report, [65] the Fe‐Nano ribbon prepared without magnetic‐field‐assisted annealing exhibits lower losses in the low‐frequency regime, which indicates lower intrinsic magnetic noise of the magnetic shielding cylinder made by the Fe‐Nano ribbon. Therefore, we avoided applying a magnetic field during the annealing process. To obtain a homogeneous Fe‐Nano ribbon with refined grain size and soft magnetic properties, the three‐step annealing method was carefully designed (Figure S10), which is guided by the differential scanning calorimetry (DSC) results of Finemet‐type amorphous ribbons [66]. This approach allows precise control of the distinct structural evolutions that occur during the annealing process. The first annealing step (pre‐annealing) was carried out at 678 to 713 K for 60 min to induce the formation of Cu clusters, which facilitates the precipitation of α‐Fe(Si) grains. No nanocrystals were formed during the first annealing process. The second annealing step was carried out in the range of 743–763 K with annealing time ranging from 30 to 45 min to increase the density of α‐Fe(Si) nucleation and further release the internal stresses originating from the quenching process. The third annealing step was conducted at 828 to 858 K for 90 min to obtain a single nanocrystalline structure. The entire annealing process was carried out under a nitrogen atmosphere.

### 4.3 Structure Characterization

To investigate the phase structure and surface morphology of the as‐quenched and annealed Fe‐based ribbons, a combination of X‐ray diffraction (XRD), scanning electron microscopy (SEM), and transmission electron microscopy (TEM) was employed. The macrostructure of as‐quenched and annealed ribbons was studied by XRD using Bruker D8 Advance with Cu‐K*α* radiation. To ensure accurate phase identification, XRD measurements of the bottom surface and top surface were performed separately. Meanwhile, SEM imaging of the as‐quenched Fe‐based amorphous ribbons was conducted using a ZEISS GeminiSEM 360. TEM observations were carried out using a JEOL JEM‐2100, enabling the acquisition of bright‐field TEM (BF‐TEM) images, high‐resolution TEM (HRTEM) images, and selected area electron diffraction (SAED) patterns to capture the structure at the nanoscale.

### 4.4 Properties Measurements

The temperature dependence of magnetization for the annealed ribbon was systematically investigated using Quantum Design MPMS 3 equipped with a high‐temperature oven. Rectangular ribbon sample with dimensions of approximately 2 mm × 2 mm was prepared and mounted in the standard non‐magnetic sample holder. The measurement was conducted under a constant applied magnetic field of 80 kA/m as the temperature was continuously increased from 300 to 975 K. To determine the Curie temperature (*T*_c_) of the annealed ribbons, the first derivative of the magnetization for temperature *dM/dT* was numerically computed. The inflection point of the (*dM*/*dT*)‐*T* curve was identified as the *T*_c_. The soft magnetic property of the annealed core with an outer diameter of 100 mm and an inner diameter of 80 mm was measured with the MATS‐3000S under a maximum applied magnetic field of 80 A/m, which was produced by Hunan Linkjoin Technology Co., Ltd. Besides, the complex permeability as a function of frequency for the annealed core was also measured with an impedance analyzer under different magnetic fields (0.02–0.8 A/m) over the range of 20–100 Hz, in order to evaluate the magnetization noise *δB*_magn_ generated by the ribbons. In our experiments, the soft magnetic property and complex permeability were measured after the cores were demagnetized and placed in a magnetic shielding environment where the residual magnetic field was reduced to the nanotesla level. The resistivity of the annealed ribbon was measured by Quantum Design PPMS.

### 4.5 Demagnetization Model

In this work, the Jiles‐Atherton (JA) model was employed to analyze the magnetization characteristics of Fe‐Nanos, thereby providing a foundation for subsequent optimization of their demagnetization performance. The parameters of the JA model are typically estimated through an iterative fitting procedure based on an experimental hysteresis loop.

To minimize the remanence, the demagnetization process was simulated by applying a degauss field through the following first‐order envelope function [67]:

(10) $$H\left(t\right)=H_{0}\left(1-t/T_{degauss}\right)\sin\left(2\pi ft\right)t\leq T_{degauss}$$

where *H*(*t*) denotes the instant magnetic field at time *t*, which varies both in amplitude and polarity throughout the demagnetization process. The initial field amplitude *H*_0_ is set as the saturation field of the material. The total demagnetization duration is denoted by *T*_degauss_. The frequency *f* determines the rate of oscillation of the sinusoidal component, and is set to 50 Hz. After demagnetization, the residual magnetic fields inside the Fe‐Nano MSC were measured by using a CTM‐6 W fluxgate magnetometer.

## Funding

This work is supported by the National Natural Science Foundation of China (NSFC) under Grant Nos. 42388101, 62403036, 62203028, 52301202, 52192602, and 52192601, China Postdoctoral Science Foundation under Grant Number 2024M754055, the Postdoctoral Fellowship Program (Grade C) of China Postdoctoral Science Foundation under Grant Number GZC20233383 and Grant Number GZC20252787, the Fundamental Research Funds for the Central Universities KG16316601, Innovation Program for Quantum Science and Technology under Grant No. 2021ZD0300501.

## Conflicts of Interest

The authors declare no conflicts of interest.

## Supporting information




**Supporting File**: advs73871‐sup‐0001‐SuppMat.docx.

## Data Availability

The data that support the findings of this study are available from the corresponding author upon reasonable request.
