# Supplementary material for: Fe‐Based Nanocrystalline Magnetic Shielding Cylinder with Subfemtotesla‐Level Magnetic Noise
Source: Adv Sci (Weinh). 2026 Jan 27;13(17):e22435. doi: 10.1002/advs.202522435 (PMC13042587; doi:10.1002/advs.202522435)
Supplement: Supplementary file 1 — Supporting File: advs73871‐sup‐0001‐SuppMat.docx. [file ADVS-13-e22435-s001.docx]

# Supporting Information

**Fe-based Nanocrystalline Magnetic Shielding Cylinder with Subfemtotesla-Level Magnetic Noise**

*Peipei Shen*, *Danyue Ma**, *Kun Wang*, *Shuang Li*, *Yanan Gao**, *Pengfei Wang*, *FuSen Yuan*, *Hua Chen*, *Zhuo Wang*, *Ziling Liu*, *Meng Xie*, *Bo Li*, *Hongbo Zhou*, *Baoan Sun**

P. Shen, D. Ma, K. Wang, S. Li, P. Wang, H. Chen, Z. Wang, Z. Liu, M. Xie, B. Li

Hangzhou Institute of Extremely-Weak Magnetic Field Major National Science and Technology Infrastructure, Hangzhou 310052, China

E-mail: [madanyue0419@buaa.edu.cn](mailto:madanyue0419@buaa.edu.cn)

D. Ma, Y. Gao

The School of Instrumentation and Optoelectronic Engineering, Beihang University, Beijing 100191, China

E-mail: [yanangao@buaa.edu.cn](mailto:yanangao@buaa.edu.cn)

D. Ma

Hefei National Laboratory, Hefei, 230088, China

F. Yuan, B. Sun

Institute of Physics, Chinese Academy of Sciences, Beijing 100190, China

E-mail: [sunba@iphy.ac.cn](mailto:sunba@iphy.ac.cn)

H. Zhou

Institute of Mechanics, Chinese Academy of Sciences, Beijing 100190, China

P. Shen and D. Ma contributed equally to this work.


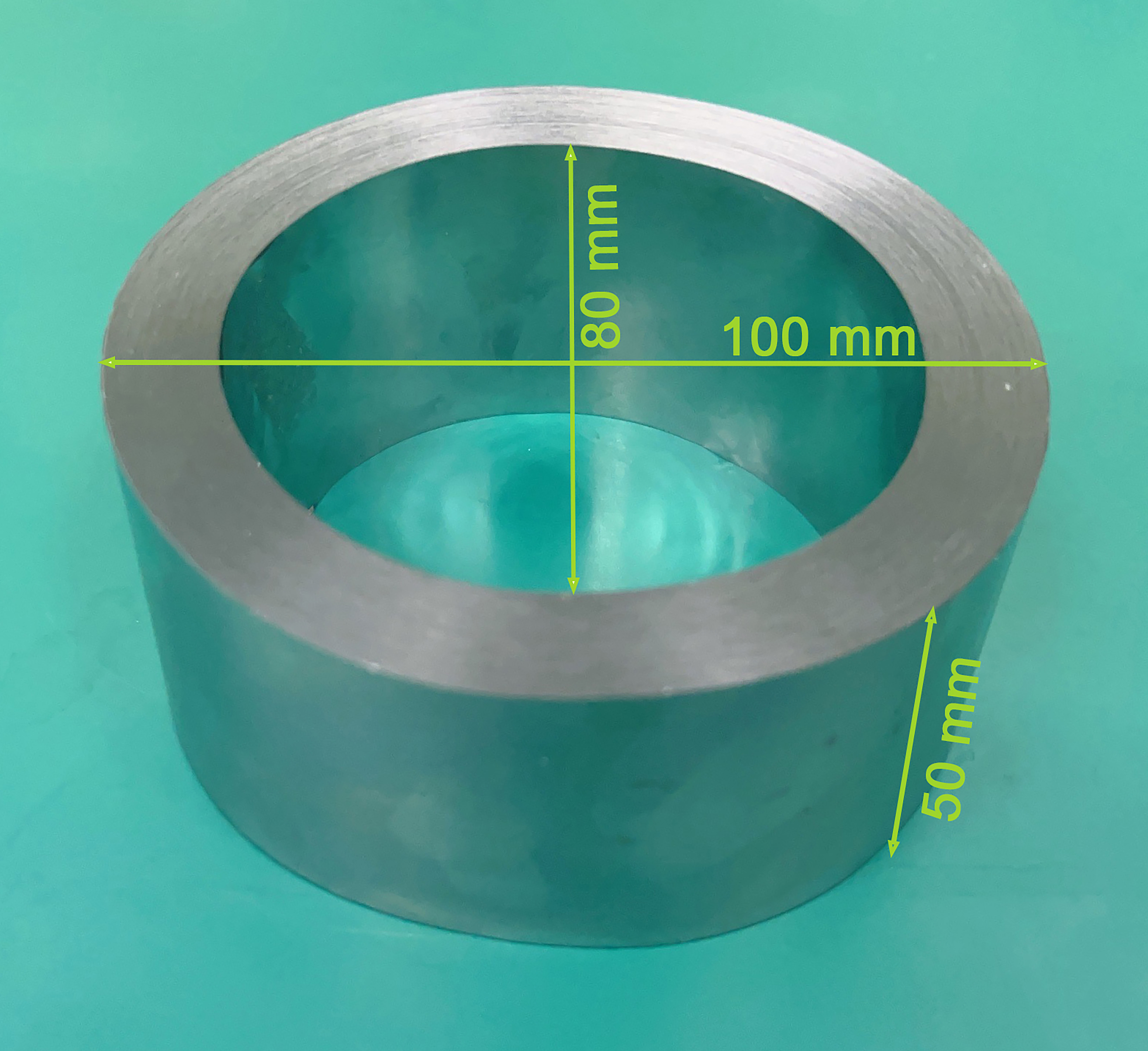


**Figure S1**. Photograph of as-quenched Fe-based amorphous alloy core (outer diameter: 100 mm, inner diameter: 80 mm, height: 50 mm).


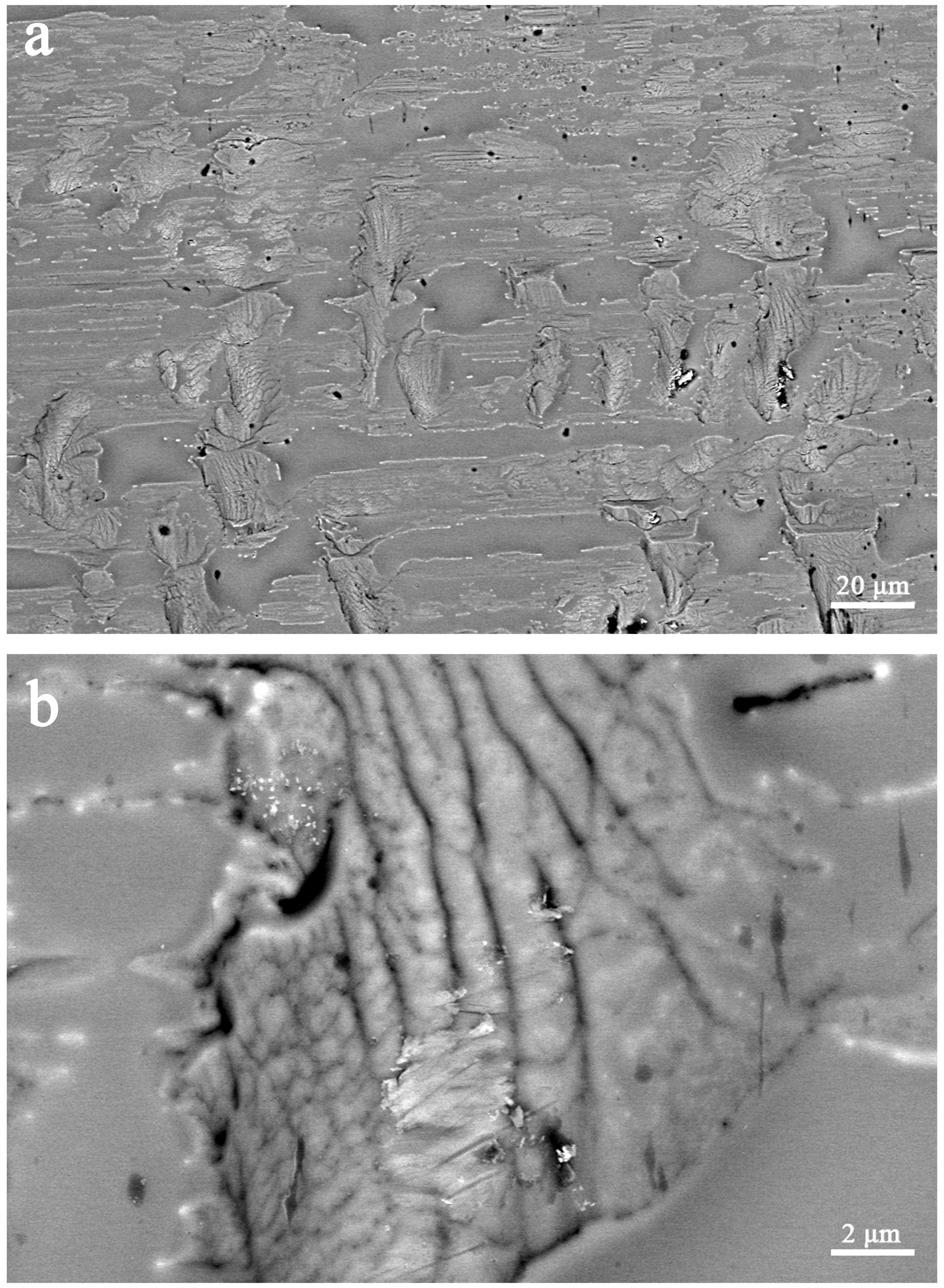


**Figure S2**. SEM images of the bottom surface of the as-quenched Fe-based amorphous ribbons with different scales.


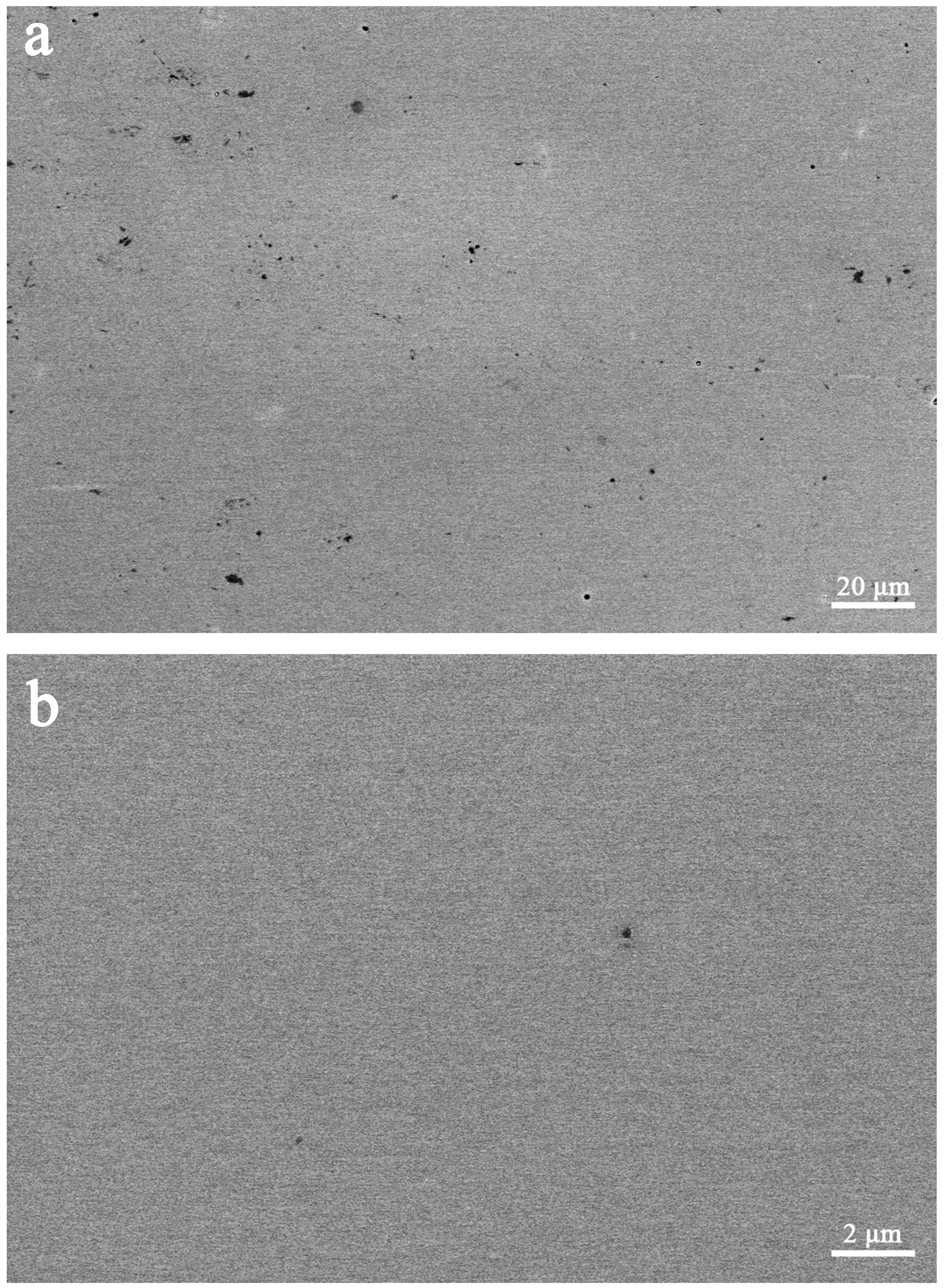


**Figure S3**. SEM images of the top surface of the as-quenched Fe-based amorphous ribbons with different scales.


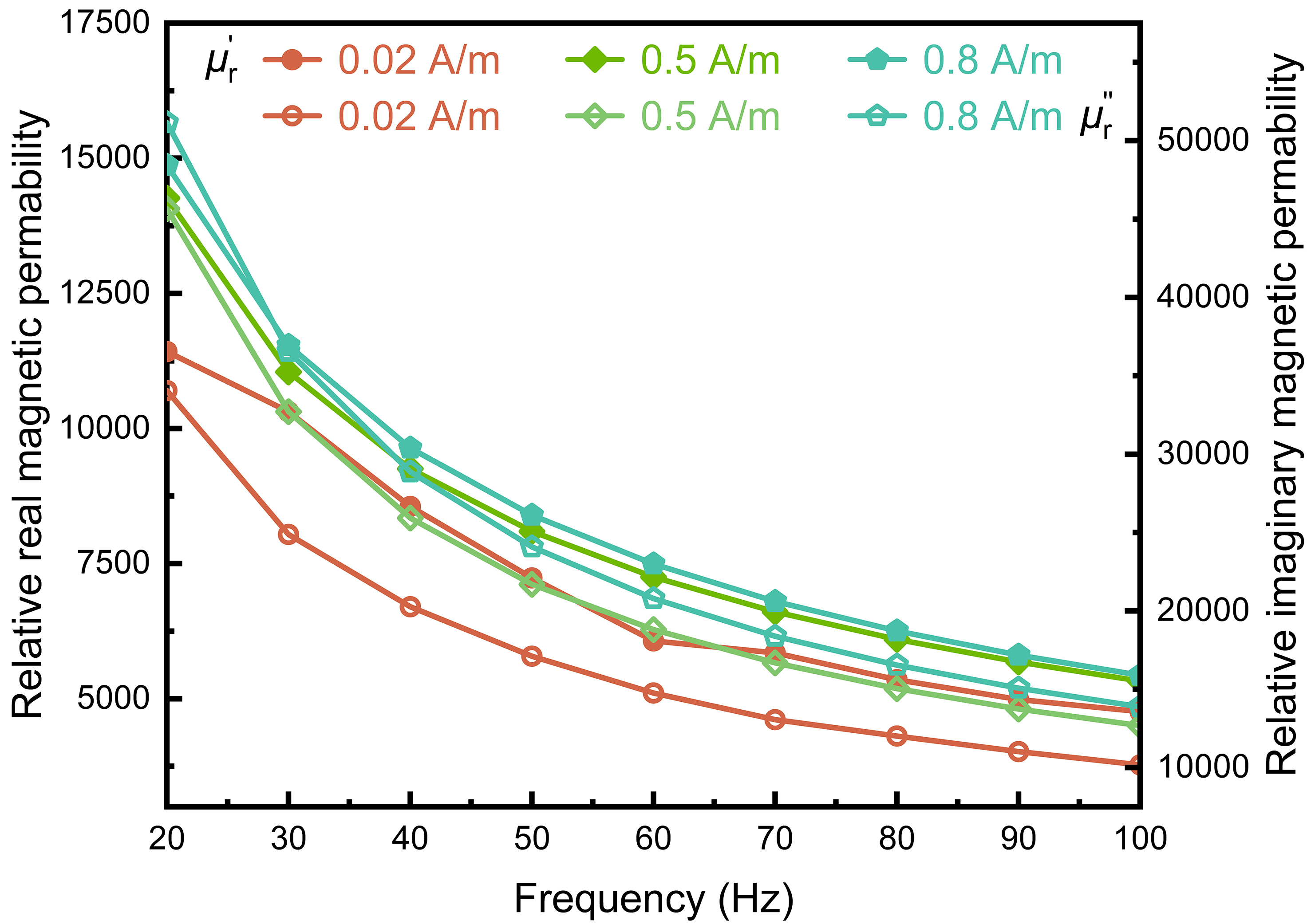


**Figure S4**. Real part ($\mu_{r}^{'}$, Left Axis) and imaginary part ($\mu_{r}^{''}$, Right Axis) of the relative complex permeability versus frequency (from 20 to 100 Hz) for *μ*-metal under different magnetic fields.


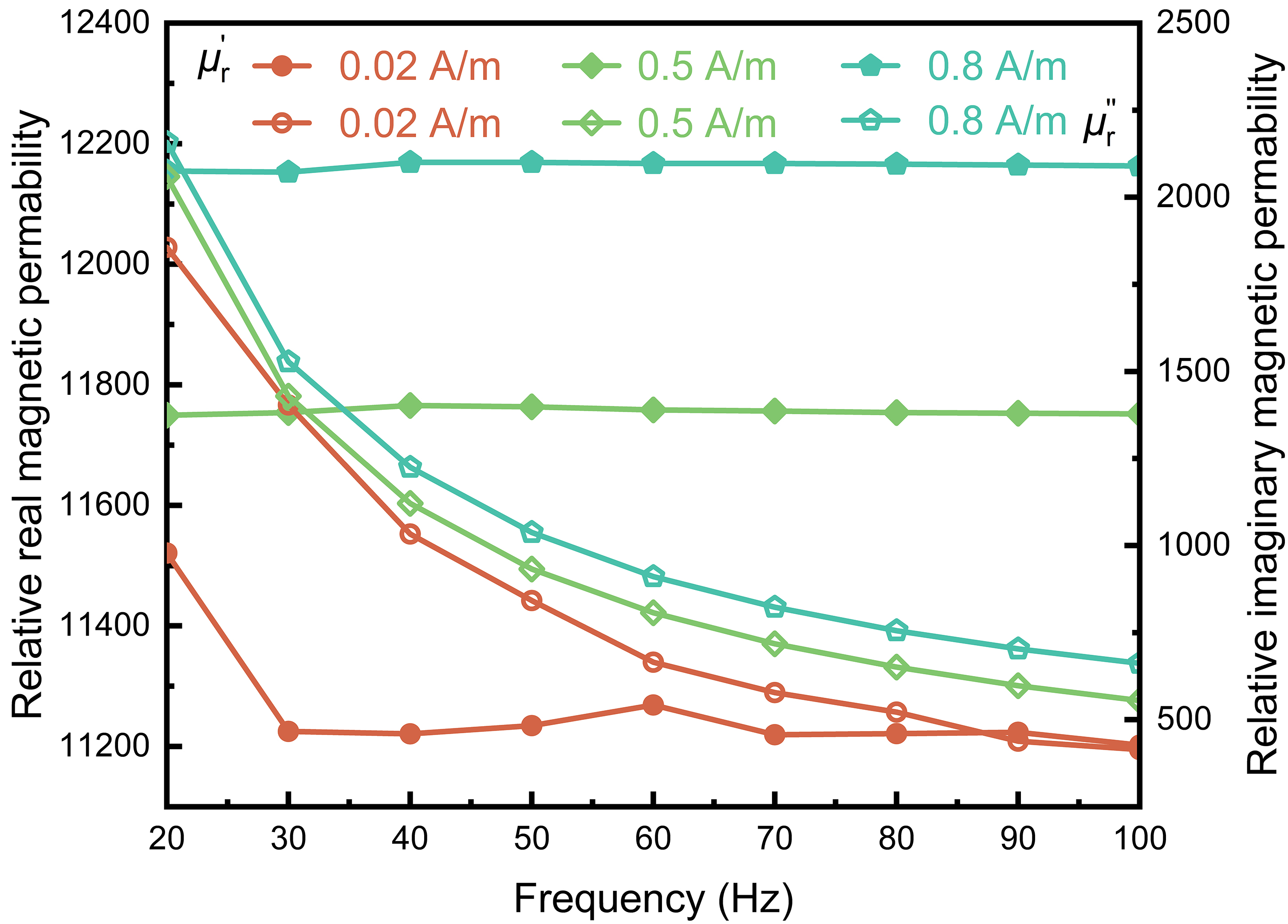


**Figure S5**. Real part ($\mu_{r}^{'}$, Left Axis) and imaginary part ($\mu_{r}^{''}$, Right Axis) of the relative complex permeability versus frequency (from 20 to 100 Hz) for Ferrite 11K under different magnetic fields.


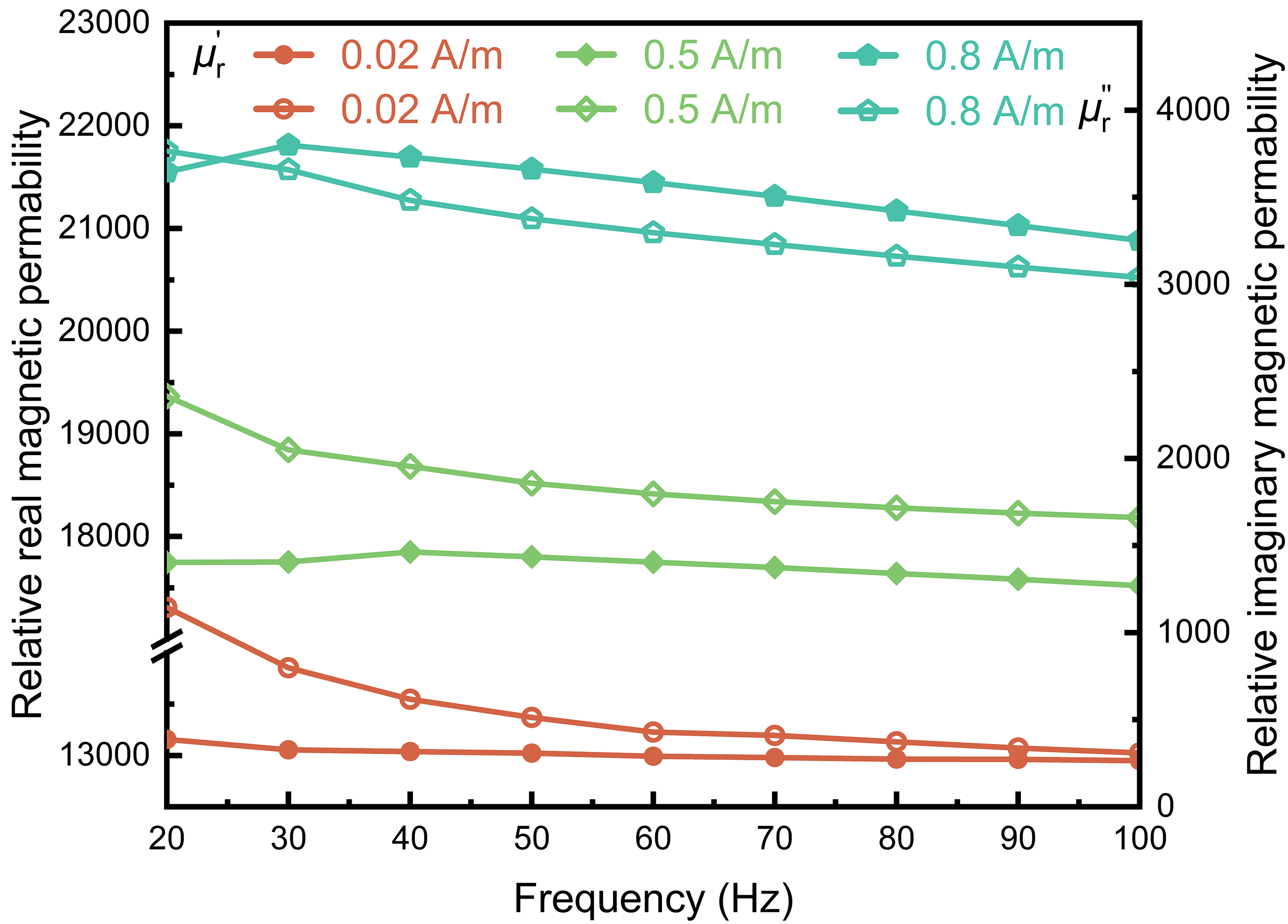


**Figure S6**. Real part ($\mu_{r}^{'}$, Left Axis) and imaginary part ($\mu_{r}^{''}$, Right Axis) of the relative complex permeability versus frequency (from 20 to 100 Hz) for Ferrite 13K under different magnetic fields.


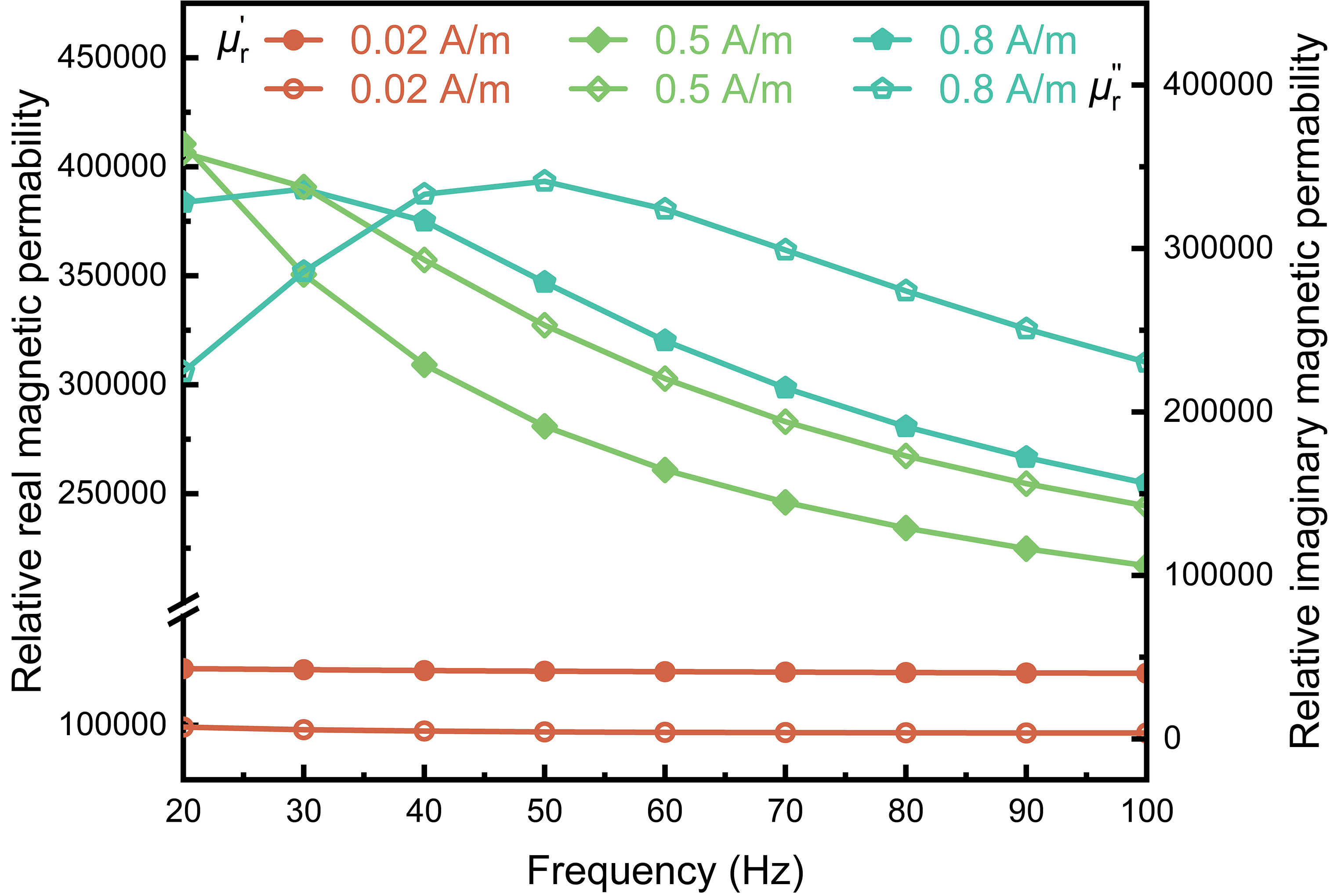


**Figure S7**. Real part ($\mu_{r}^{'}$, Left Axis) and imaginary part ($\mu_{r}^{''}$, Right Axis) of the relative complex permeability versus frequency (from 20 to 100 Hz) for Co-based amorphous alloy under different magnetic fields.





**Figure S8**. Photograph of four Fe-Nano MSCs with different thicknesses (inner diameter: 240 mm, height: 390 mm). From left to right: single-layer Fe-Nano MSC, three-layer Fe-Nano MSC, five-layer Fe-Nano MSC, and ten-layer Fe-Nano MSC. All Fe-Nano MSCs were with demagnetization coils.


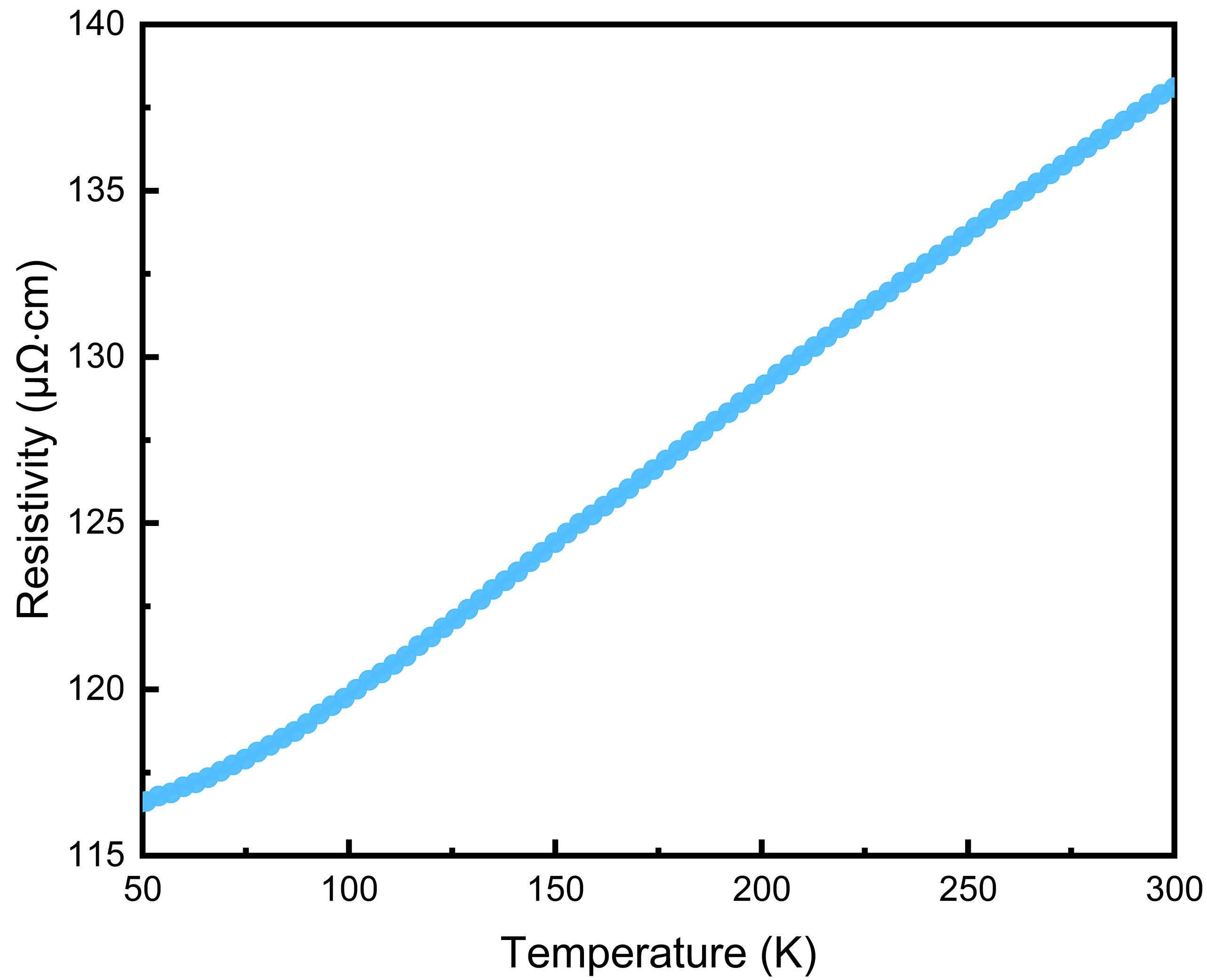


**Figure S9**. Temperature dependence of the electrical resistivity *ρ*(*T*) of the Fe-based nanocrystalline ribbon.


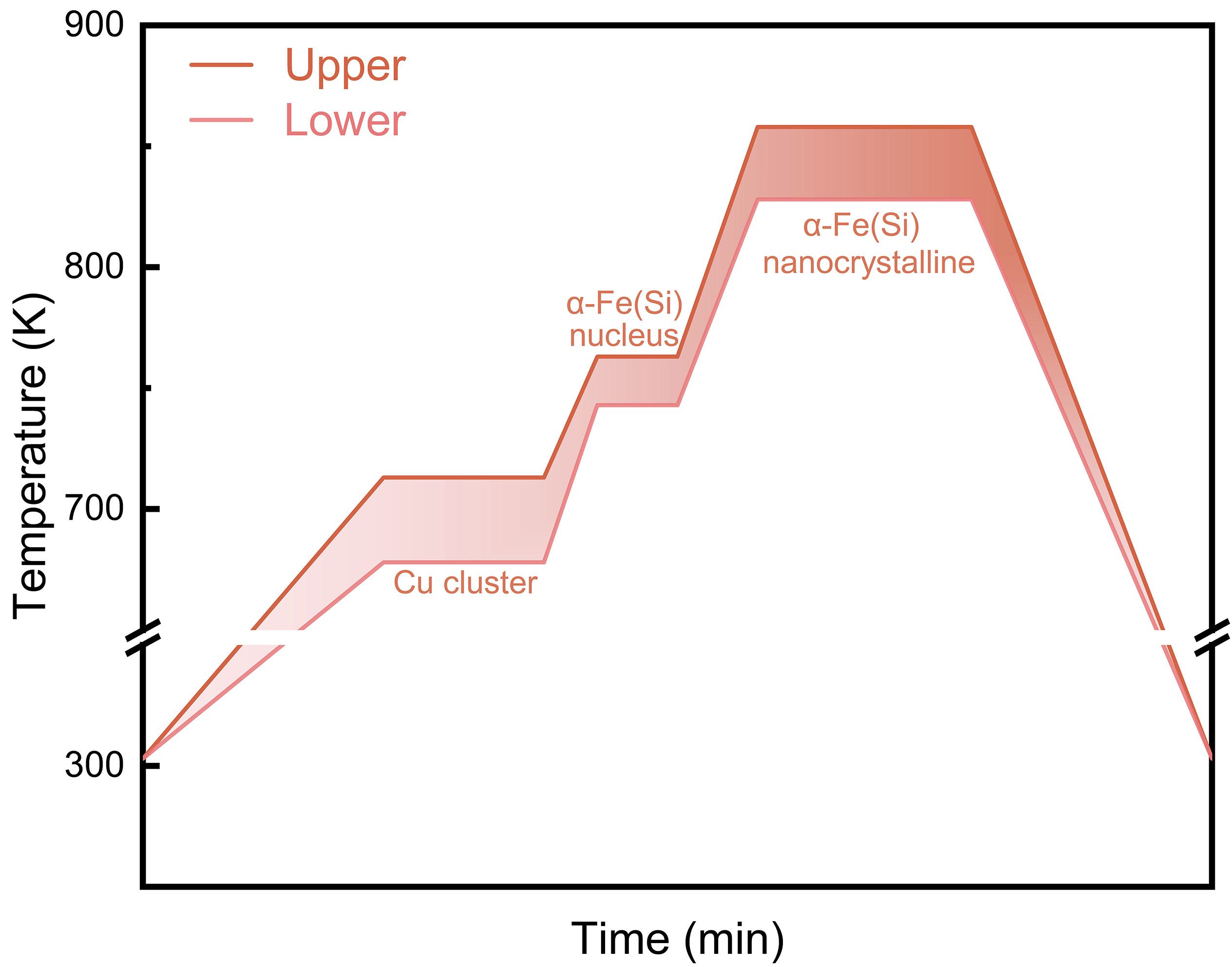


**Figure S10**. The annealing process curves.
